# A longitudinal prospective study investigating serial measurements of plasma neutrophil gelatinase-associated lipocalins (NGAL) for the prediction of chronic kidney disease and acute kidney injury in emergency department patients

**DOI:** 10.1186/s12882-026-04791-7

**Published:** 2026-01-30

**Authors:** Vicky Jenny Rebecka Wetterstrand, Thomas Kallemose, Jesper Juul Larsen, Lennart Friis Hansen, Lisbet Brandi

**Affiliations:** 1 Department of Endocrinology and Nephrology, North Zealand University Hospital, Hillerød, Denmark; 2 Department of Clinical Research, Copenhagen University Hospital Amager and Hvidovre, Hvidovre, Denmark; 3 Department of Emergency, North Zealand University Hospital, Hillerød, Denmark; 4 Department of Clinical Biochemistry, Odense University Hospital, Odense, Denmark; 5 Institute of Clinical Medicine, University of Copenhagen, Hillerød, Denmark

**Keywords:** NGAL, CKD, AKI, Emergency department, Nephrology, Prospective study

## Abstract

**Background and hypothesis:**

Acute kidney injury (AKI) and chronic kidney disease (CKD) are associated with high morbidity. Current diagnostic markers, e.g., creatinine (Cr) and estimated glomerular filtration rate (eGFR), lack precision in early kidney disease detection. Neutrophil gelatinase-associated lipocalin (NGAL) has been proposed as a more sensitive biomarker for kidney injury. This study examined the effectiveness of plasma NGAL (pNGAL) as biomarker for CKD and AKI prediction in an emergency department (ED) setting and for predicting the presence of CKD and AKI in future admissions (readmission). The performance was compared to that of C-reactive protein (CRP).

**Methods:**

A prospective longitudinal study was conducted at North Zealand University Hospital in Denmark, including 925 patients admitted to the ED. pNGAL and CRP were measured at admission, with follow-up pNGAL measurements for hospitalized patients. CKD and AKI were defined according to KDIGO guidelines. Receiver operating characteristic (ROC) analyses assessed the sensitivity, specificity, and predictive value of pNGAL and CRP, for AKI and CKD, at both admission and readmission.

**Results and conclusion:**

pNGAL is useful for ruling out AKI and CKD, especially at admission, but its low PPV limit its diagnostic accuracy, particularly at readmission. It is more effective as a screening tool rather than a standalone diagnostic marker. Higher predictive performance was observed for pNGAL compared to CRP. For serial pNGAL measurements, CKD patients exhibited higher initial levels at admission, but these differences diminished by day four, with considerable variability observed thereafter.

**Supplementary Information:**

The online version contains supplementary material available at 10.1186/s12882-026-04791-7.

## Introduction

Kidney disease has risen from the 10th to the 9th leading cause of death worldwide within just a few years. Mortality continues to increase, from 813,000 deaths in 2000 to 1.3 million in 2019 according to WHO [1]. Survivors of acute kidney injury (AKI) have a substantially higher risk of developing end-stage renal disease (ESRD). In the past two decades, a bidirectional relationship between AKI and chronic kidney disease (CKD) has been recognized, AKI is a risk factor for CKD, while pre-existing CKD increases the risk of AKI tenfold [2, 3]. Both are associated with high morbidity, including cardiovascular disease [3], emphasizing the importance of early detection before the onset of kidney dysfunction.

Exogenous filtration markers such as inulin and iohexol provide accurate measures of glomerular filtration rate (GFR) [4] but are time- and cost-intensive. Therefore, the KDIGO guidelines [5] recommend using endogenous biomarkers such as creatinine (Cr) to estimate GFR (eGFR). However, eGFR based on Cr is imprecise at higher GFR levels (≥ 60 mL/min/1.73 m²) and varies with sex, muscle mass, and diet [5]. This makes Cr suboptimal for diagnosing AKI and CKD, particularly in the elderly or malnourished. Other functional biomarkers, including plasma cystatin C [6, 7], blood urea nitrogen (BUN) [8, 9], and urine albumin [10, 11], are also limited by non-renal factors. Thus, there remains a need for improved diagnostic methods for kidney disease.

Damage biomarkers provide earlier and more specific information on kidney injury by reflecting cellular stress and injury rather than global renal function [10, 11]. Several such biomarkers, neutrophil gelatinase-associated lipocalin (NGAL) [12], kidney injury molecule-1 (KIM-1) [10], tissue metalloproteinase-2 (TIMP-2) [13], insulin-like growth factor binding protein 7 (IGFBP7) [10, 13], and growth differentiation factor 15 (GDF15) [14], have been identified, though their clinical adoption is limited by cost, availability, or specificity. Integrating functional and damage biomarkers, as recommended by the 23rd Acute Disease Quality Initiative (ADQI) [15], may improve AKI diagnosis and risk stratification. Among these, the iron-binding 25 kDa lipocalin NGAL has emerged as a promising candidate [12].

Neutrophil granulocytes release NGAL, and its expression is regulated by inflammatory mediators such as IL-1β, IFN-γ, and other cytokines [12, 16, 17]. Plasma NGAL (pNGAL) levels rise rapidly within 2–6 h after kidney injury [12], whereas serum creatinine (sCr) typically increases only after 48–72 h [11]. In AKI, pNGAL elevation is driven by increased tubular synthesis and systemic inflammation, while in CKD it reflects reduced clearance due to chronic filtration impairment [12]. Consequently, elevated pNGAL can indicate both acute and chronic kidney damage.

C-reactive protein (CRP), a well-established inflammatory marker, is also relevant to renal disease. Besides systemic inflammation, CRP can be locally produced by kidney cells during injury [18], and high CRP levels are linked to increased risk of AKI and CKD, particularly in sepsis or myocardial infarction [19–21]. Comparing CRP and pNGAL levels may therefore help distinguish the inflammatory and structural components of kidney injury in the emergency department (ED).

Earlier ED-based studies on pNGAL as a predictor for AKI and/or CKD have used heterogeneous populations and definitions [22–25]. Recent investigations [26–28] remain limited to specific ED subgroups, resulting in wide reported cutoffs, 129–490 ng/mL for AKI [22, 23, 26–28] and 104–390 ng/mL for CKD [23, 25, 29, 30], and no consensus on optimal thresholds. Few have explored serial pNGAL measurements [31–33], and only one ED study [25] has examined pNGAL’s prognostic value for CKD progression, finding no predictive improvement. Moreover, assay variability in detecting NGAL’s molecular forms (monomeric, dimeric, heterodimeric) further complicates clinical interpretation. As the monomer form is released by renal tubular cells in AKI and CKD, while the dimeric and heterodimeric complexes with MMP-9 are produced by neutrophils, this lack of antibody selectivity in the assays leads to cross-reactivity, which reduces the clinical accuracy of NGAL measurements [34, 35].

Our recent study [36], a sub-cohort of the present population, evaluated KDIGO-listed risk factors for AKI and found that traditional exposures and susceptibilities alone were poor predictors. Adding pNGAL and CRP significantly improved prediction accuracy, with pNGAL providing the greatest benefit. Building on these findings, the current study aims to assess pNGAL’s ability to identify CKD in the ED, validate its role as an AKI rule-out biomarker [37], and explore whether CRP and serial pNGAL testing enhance early detection and monitoring of kidney injury progression.

## Methods

### Study design and setting

This prospective longitudinal study was conducted at North Zealand University Hospital (Denmark) [38] enrolling patients from October 3, 2019, to December 26, 2020. Patients ≥ 18 years admitted to the ED during three selected days each month were included, excluding obstetric and trauma patients.

**Fig. 1:**
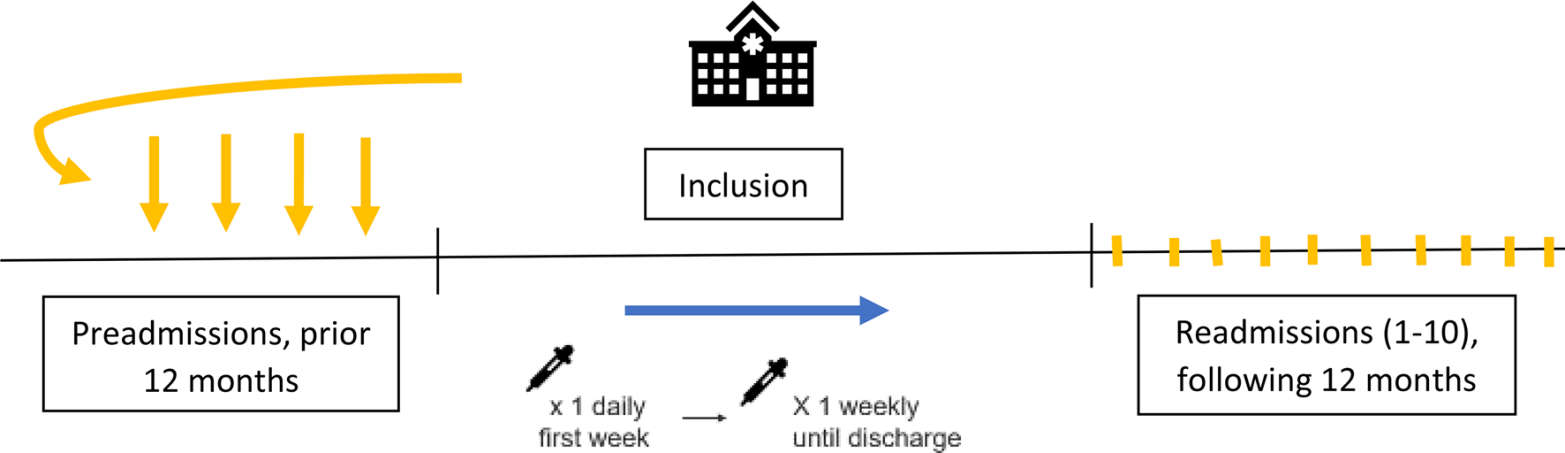
Timeline over study inclusion. Patients were consecutively enrolled through the Emergency Department for three days each month over a 15-month period. Blood samples for pNGAL were collected daily during the first week, then weekly until discharge. Data on diagnosis, medication, and laboratory results were retrieved from medical records, covering up to one year before inclusion and a one-year follow-up period (maximum up to 10 readmissions)

Blood samples were collected within the first hour of admission at enrollment, including standard ED tests, pNGAL, and biobank samples. For hospitalized patients, daily pNGAL samples were taken for a week, followed by weekly sampling. Discharged patients were included but without further pNGAL samples. Kidney function was assessed up to one year before and one year after admission via medical records (Fig. 1). Admissions during the enrollment period were considered first-time admissions, whereas any subsequent hospitalizations within one year were registered as readmissions.

pNGAL samples were processed within 120 min from collection, initially frozen at − 20 °C, and then transferred weekly to a − 80 °C freezer. As patient enrollment occurred three days per month, all samples were transferred within three days of collection, consistent with the manufacturer’s recommendations for NGAL stability (BioPorto, Denmark) [39], which indicate stability for 1 day at room temperature, up to 3 days at 2–8 °C, and up to 1 year at ≤–70 °C, with tolerance for up to three freeze–thaw cycles. Analyses were performed between October 1, 2019, and January 1, 2021, using the NGAL ST001 assay (measuring range 50–3000 µg/L, CV = 14%) [39] on a Siemens Dimension Vista 1500. This handling procedure ensured that biomarker integrity was maintained throughout storage and processing.

### Data collection

The data collected through medical records (Sundhedsplatformen (SP), Epic, Verona, Wisconsin) included medical diagnosis, age, gender, pharmaceutical medication, vital parameters and laboratory test results. pNGAL results were retrieved from the hospitals laboratory information system (LABKA II) database. All data was merged using the Danish unique Central Personal Registry (CPR) number into the secure web-based Research Electronic Data Capture (REDCap) (REDCap, Fort Lauderdale, Florida)-database.

### Definition of AKI and CKD

The patients were divided into non-AKI, AKI and/or CKD according to the KDIGO [40] guidelines at admission and/or readmission. The identification of one or more kidney conditions during readmission was considered irrespective of the specific timing, provided it occurred within a 12-month period from the point of initial inclusion. In accordance with the KDIGO [18] definition of acute kidney injury (AKI), we defined AKI as an increase in plasma creatinine (pCr) of ≥ 26.5 µmol/L from the mean baseline pCr value measured during the preceding twelve months, excluding the 7 days prior to admission (-7 to -365 days). This mean value was considered to represent each patient’s habitual (baseline) kidney function. Because the admission pCr value was obtained in the ED during an acute presentation, any elevation from baseline was assumed to reflect an acute change consistent with AKI. We acknowledge the importance of distinguishing transient AKI (< 48 h), persistent AKI (> 48 h to < 7 days), and acute kidney disease (AKD, 7–90 days) [5]; however, our analysis focused on AKI identified at the time of hospital admission and did not subclassify cases by duration. To define CKD, we used a decreased eGFR for at least three months with KDIGOs GFR category G3 upper limit (GFR < 60 ml/min/1.73 m^2^) as a minimum [40]. The pNGAL measurement obtained at admission was used to represent the patient’s acute kidney function at the time of arrival. Conversely, the final NGAL measurement before discharge was considered indicative of kidney status following treatment during hospitalization. Accordingly, the admission pNGAL value was analyzed to evaluate its predictive ability for AKI and CKD at presentation. The discharge NGAL value was examined in relation to the likelihood of developing AKI or CKD at a future readmission within one year, based on the premise that persistently elevated NGAL levels at discharge may signal ongoing or unresolved kidney stress.

### Outcomes

Multiple outcomes for AKI and CKD are analyzed in the study, these being the standalone conditions AKI at admission, CKD at admission, AKI at readmission and CKD at readmission and the composite conditions (non-AKI/non-CKD, only AKI, only CKD and AKI + CKD) at admission. The standalone conditions are defined from the AKI and CKD definitions described in the previous section at the specified timepoints, with each condition being evaluated separately. Whereas the composite conditions evaluate the conditions from an ordered perspective going from non-AKI/CKD to AKI + CKD. Additionally, the composite condition requires data to be available for both AKI and CKD evaluation, whereas the standalone condition only require data for evaluation of their respective condition. In this study, “admission” refers to the patient’s initial hospital admission through the ED during which they were included in the study. “Readmission” is defined as any subsequent hospital admission, across all departments, occurring within a 12-month period following discharge from the ED.

### Ethics

The Danish ethical regulations were followed and approved by the Danish National Committee (J.nr.H-19003424) and the Danish Data Protection agency (VD-2019-143).

### Consent

All patients participating in the study had a written consent, in accordance with the Danish ethical committee.

### Statistical method

Descriptive statistics were presented as mean with standard deviation (SD) or as frequencies with percentages.

pNGALs ability to predict AKI and CKD were analysed using Receiver operating characteristic (ROC) analyses, including only pNGAL as predictor in the analysis. The standalone conditions were analysed in separate models for each condition, and the composite conditions as a separate model. For the composite conditions a cumulative ROC analysis was performed, with an assumed increased severity in condition (non-AKI/non-CKD < AKI only < CKD only < AKI +CKD). An ordered logistic regression with the composite conditions as dependent variable and pNGAL as independent were fitted, but the proportionality, evaluated by the brant test [41], could not be assumed. Therefore, the analysis was done as separate ROC analysis with cases consisting of three increasing sets of conditions AKI only or worse (AKI only or AKI in CKD), CKD only or worse (CKD only or AKI in CKD) and AKI + CKD (AKI in CKD). For the prediction at admission the first pNGAL measurement during hospitalization was used and for readmission the last NGAL measure was used to reflect a treated and soon to be discharged patient.

These analyses were repeated for the standalone conditions with CRP instead of pNGAL. Estimates for optimal cut-off values, based on Youden index, from the ROC analyses were presented as sensitivity, specificity, positive predictive value (PPV) and negative predictive value (NPV), additionally ROC curves with AUC are also presented.

Serial changes in pNGAL were calculated as difference between first and last pNGAL measurement, and categorized into decrease, unchanged and increase. Sensitivity, specificity, PPV and NPV were estimated for cut-offs for Increase or unchanged vs. decreased and increase vs. unchanged or increase when prediction AKI and CKD at readmission.

Pearson correlation coefficient was estimated between pNGAL and CRP and between pNGAL and number of readmissions, both for all patients and only AKI patients. All analyses were done using R version 4.1.0 [42].

## Results

### Patient evaluation for AKI and CKD at admission and readmission

**Fig. 2:**
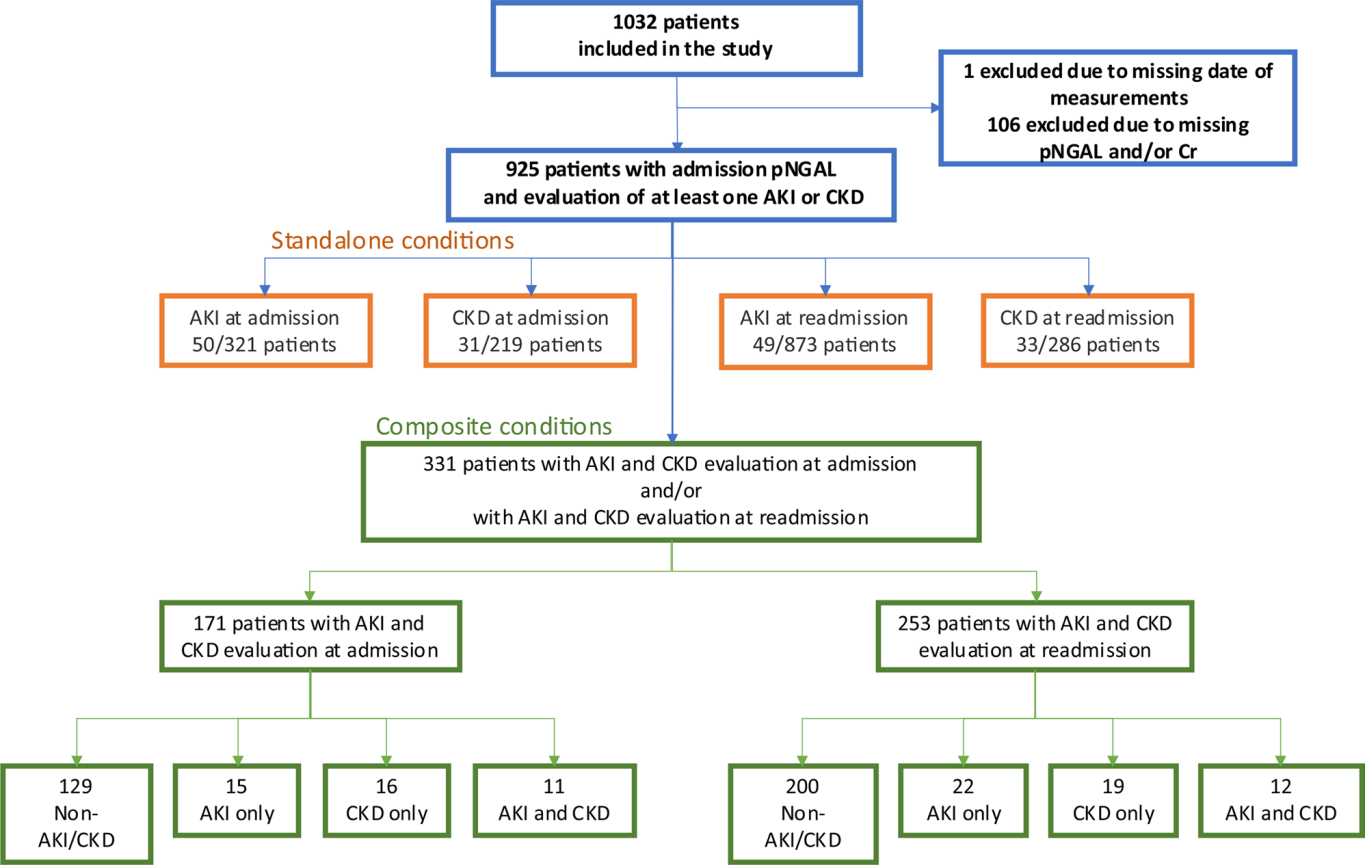
Flowchart over patient inclusion and distribution of diagnostic status at admission and readmission. In the 925 included patients it was possible to evaluate one or more potential diagnosis (AKI, CKD) at admission and/or readmission for the standalone conditions. For evaluation of composite conditions 331 had an initial pNGAL measurement along with the required data for evaluating both AKI and CKD at either admission, readmission, or both. Among these, 171 patients were evaluated at admission, and 253 at readmission. Patients were categorized into four groups based on their diagnostic status: healthy, AKI only, CKD only, or concurrent AKI and CKD, evaluated at either admission and/or readmission

1,032 patients were enrolled. After excluding 107 with incomplete data, 925 remained. AKI at readmission was assessed in 873 patients, identifying 49 cases (standalone condition). CKD at readmission (standalone), AKI at admission (standalone), and CKD at admission (standalone) were evaluated in fewer patients (219 to 321) but had similar condition counts (31 to 50) (Fig. 2).

**Fig. 3:**
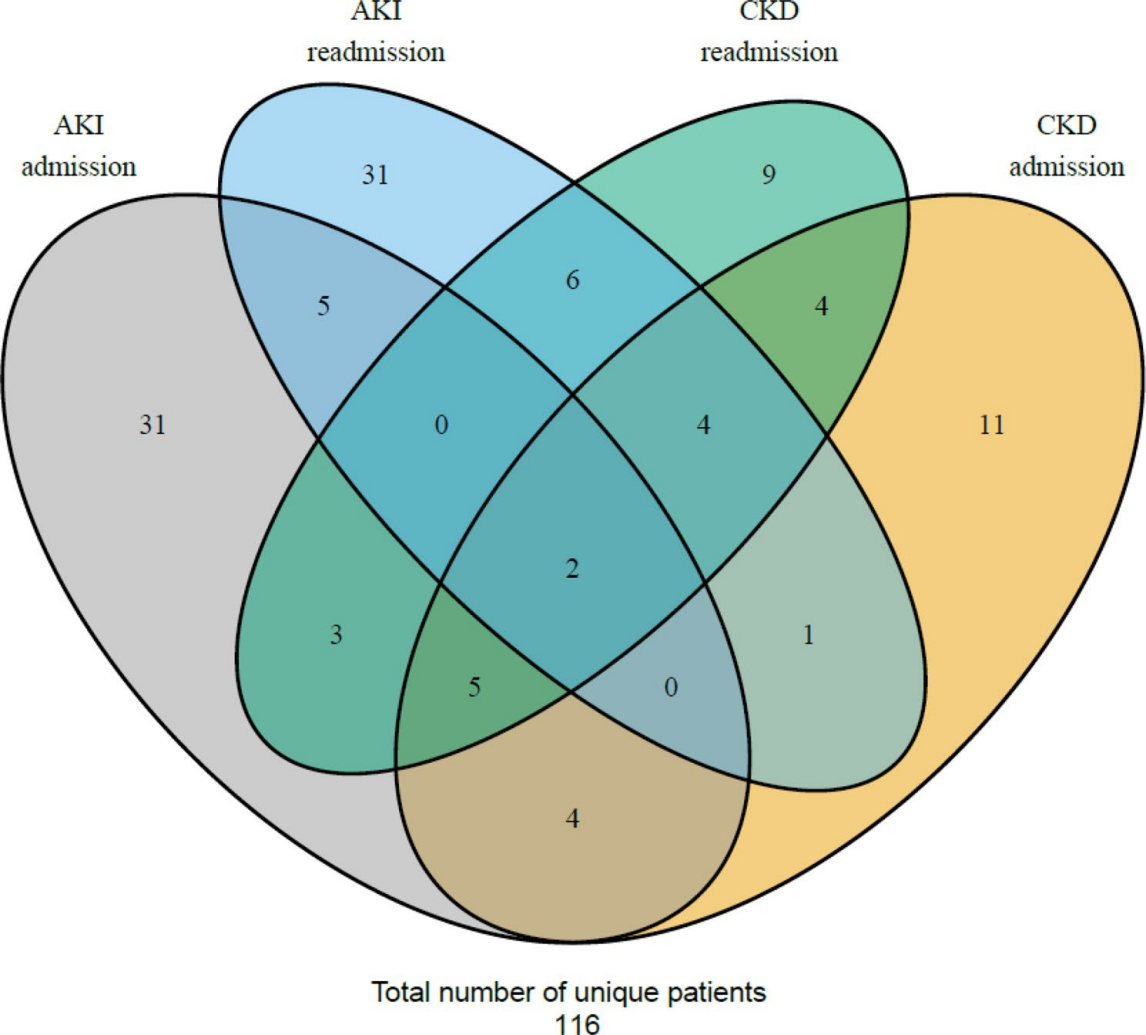
Venn diagram identifying AKI and CKD distribution (standalone conditions) and their combinations. The Venn diagram identifies the number of patients (derived from standalone conditions) with different combinations of diagnosis, in patients with or without overlapping diagnosis, in the four categories. The four colored ellipse in the Venn diagram represents AKI _admission_ (gray), AKI _readmission_ (blue), CKD _readmission_ (green) and CKD _admission_ (yellow). There are in total 116 unique patients. The included patients in the Venn diagram didn’t require data for evaluation of both AKI and CKD and didn’t require data for both admission and readmission

In total, 116 patients had at least one condition, 34 with two or more (Fig. 3). Composite condition data was available for 331 patients: 171 at admission and 253 at readmission. Most had no AKI/CKD at both points, with similar distributions for AKI, CKD, and AKI + CKD at admission (11–15 cases). At readmission, AKI + CKD was slightly less frequent [12] than AKI only [22] and CKD only [19] (Fig. 2).

**Fig. 4:**
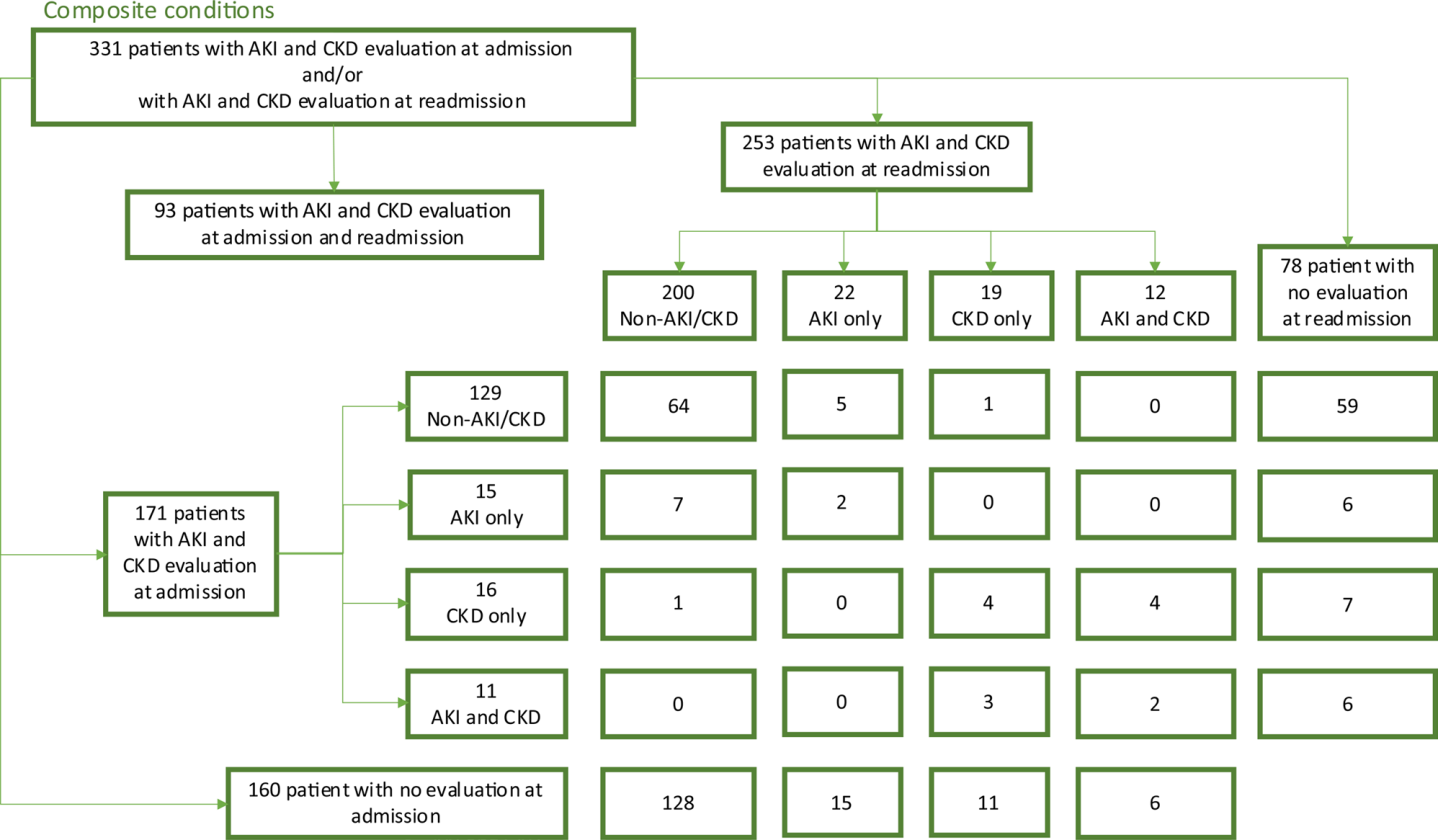
Flowchart over composite conditions. Among the 331 patients, 171 were evaluated at admission, and 253 at readmission. 93 patients were evaluated for AKI and CKD at both admission and readmission. Patients were categorized into four groups based on their diagnostic status: healthy (non-AKI/CKD), AKI only, CKD only, or concurrent AKI and CKD. As shown in the flowchart, some patients had incomplete data, missing at either admission or readmission

Among 93 patients assessed at both admission and readmission, 72 had no change, 10 worsened, and 11 improved (Fig. 4).

Of 915 patients, 516 (56%) were female, with a median age of 60 years. At ED admission, 0.3% had kidney transplants, and 4.9% had prior CKD diagnoses (Table 1).

**Table 1:** Demographics, disease, and laboratory characteristics of the patients at ED admission

Parameters	
Total number of patients, n (% female)	925 (55.8%)
Age, median years (IQR)	60 (44: 74)
Kidney transplant, n (%)	2 (0.3%)
Diabetes, n (%)	71 (11.8%)
Cancer*, n (%)	60 (10%)
Anemia^, n (%)	23 (3.8%)
CKD#, n (%)	29 (4.9%)
Cardiovascular disease, n (%)	75 (8.1%)
Lung disease, n (% of total)	109 (11.8%)
Liver disease, n (%)	13 (1.4)
Hgb (mmol/L), median (IQR)	8.6 (7.9: 9.2)
pCr (µmol/L), median (IQR)	73 (61: 86)
ALAT (U/L), median (IQR)	22 (15: 34)

Abbreviation: ED= emergency department; n= number of patients; IQR=interquartile range; CKD= chronic kidney disease

*All patients diagnosed with any type of cancer form, documented in medical journal. First excluded when relapse-free ≥ 5 years

^ Hgb < 7mmol/L for female and <8mmol/L for male ≥ 3 months

#All patients recorded with the diagnose CKD in medical journal

### pNGAL cutoff values for composite condition

**Fig. 5:**
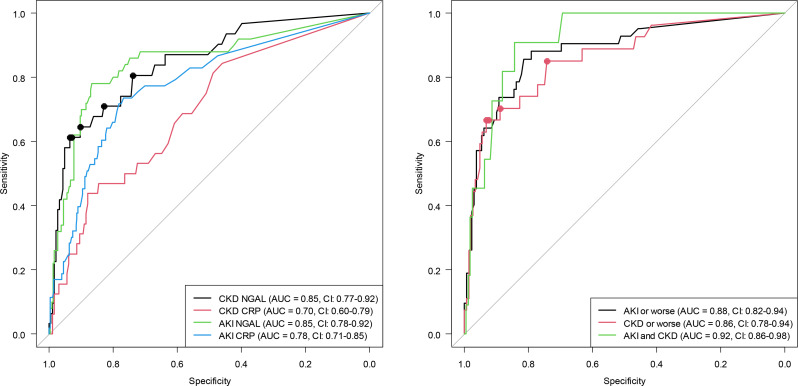
Receiver Operating Characteristic (ROC) for NGAL and CRP performance for the diagnosis of AKI and CKD at admission. Receiver Operating Characteristic (ROC) were used to calculate area under the curve (AUC) and 95% confidence intervals (CI) for the diagnosis of acute kidney injury (AKI) and chronic kidney disease (CKD) based on pNGAL and CRP respectively, at admission and AKI or worse (AKI only, CKD only or AKI and CKD), CKD or worse (CKD only or AKI and CKD) and AKI and CKD based on pNGAL at admission. Dots indicate cutoff values that all are within 0.01 units of the optimal cutoff on the Youden index

For the composite condition, the pNGAL cutoff was 79 ng/mL for patients with AKI-only or worse, 83 ng/mL for CKD or worse and 131 ng/mL for both AKI + CKD. All cutoffs had high levels of sensitivity and specificity (79–91%), NPV (95–99%), but poor PPV, with lower values the worse the condition. ROC curves from the analysis are presented in Fig. 5 (Table 2).

**Table 2:** pNGAL performance at optimal cutoff values

Kidney status	NGAL cutoff (ng/mL)	Sensitivity (%)	Specificity (%)	PPV (%)	NPV (%)
**Standalone condition**					
CKD admission	131	65 (48-81)	90 (86-95)	53 (37-69)	94 (90-97)
AKI admission	111	78 (67-89)	87 (83-91)	52 (41-63)	96 (93-98)
CKD readmission	69	94 (86-100)	64 (59-70)	26 (18-33)	99 (97-100)
AKI readmission	81	67 (54-80)	75 (72-77)	14 (9-18)	97 (96-99)
**Composite conditions**					
AKI + admission	79	88 (78-98)	79 (72-86)	58 (46-70)	95 (91-99)
CKD + admission	83	85 (72-99)	74 (67-81)	38 (26-51)	96 (93-100)
AKI + CKD admission	131	91 (74-100)	84 (79-90)	29 (14-44)	99 (98-100)

Receiver Operating Characteristic (ROC) were conducted to analyze NGAL performance and calculate NGAL cutoff value for AKI and CKD at admission and readmission, respectively. AKI +: AKI or worse (AKI only, CKD only or AKI + CKD), CKD +: CKD or worse (CKD only or AKI + CKD)

### pNGAL cutoff values for standalone conditions

pNGAL cutoff at admission was estimated at 111 ng/ml for AKI and 131 ng/ml for CKD. Cutoffs had similar specificity (around 90%) and PPV (around 50%), AKI had a bit larger sensitivity at 78% compared to 65% for CKD. For readmission cutoffs were estimated at 81 and 69 ng/ml for AKI and CKD respectively. These cutoffs had specificity of 64% and 75%, PPV of 14% and 26% and sensitivity of 67% and 94% for AKI and CKD respectively. Cutoffs for AKI and CKD for both admission and readmission had high NPV (96–99%) (Table 2).

### Cutoff robustness

When looking at the curvature of the ROC curves from the analysis (Fig. 5) CKD at admission has very low curvature in the center, suggested that multiple cutoffs might have similar Youden index value for CKD at admission. Further inspection of the values revealed five cutoffs with a Youden index value within 0.01 units of each other, with pNGAL values for the cutoffs being between 83 and 158 ng/ml, with lower cutoffs having higher sensitivity and higher cutoffs having higher specificity. A similar but lesser pattern was observed for the CKD or worse analysis, and the value were therefore also investigated further here. This revealed four cutoffs, also with Youden index values within 0.01 of each other. Also, these with pNGAL cutoff values between 83 and 158 ng/ml, and again with lower cutoffs having higher sensitivity and higher cutoffs having higher specificity. Additionally, if the cutoff value for CKD readmission is used in the CKD admission analysis, values of 87 for sensitivity and 62 for the specificity are estimated, and if the cutoff value for CKD admission is used in the CKD readmission analysis, values of 65 for sensitivity and 90 for the specificity are estimated.

### Serial pNGAL measurements

Most patients (71%) had only a single pNGAL measurement, as they were discharged from the ED within 24 h; 12% had two measurements, and 17% had three or more. The predictive performance of the serial pNGAL analysis were all adequate at best except for NPV which had values between 85% and 91%. However, all values were lower than the corresponding first or last pNGAL analysis (Table 3). At admission, CKD patients initially had higher pNGAL values than non-CKD patients, which plateaued by day four, after which the trends appeared similar across groups. Overall, changes in pNGAL levels during admission did not show a clear pattern and exhibited considerable variation among patients (supplementary Figs. 1 and 2).

**Table 3:** Performance of serial changes in pNGAL

Kidney status at readmission	Serial changes in pNGAL	Sensitivity (%)	Specificity (%)	PPV (%)	NPV (%)
CKD	Increase or unchanged	67 (43–91)	34 (24–44)	15 (7–24)	85 (73–97)
CKD	Increase	60 (35–85)	55 (45–66)	20 (8–31)	88 (80–97)
AKI	Increase or unchanged	54 (35–73)	41 (35–48)	9 (5–14)	89 (83–95)
AKI	Increase	46 (27–65)	63 (57–69)	12 (6–19)	91 (87–96)

Receiver Operating Characteristic (ROC) were conducted to analyze serial changes in NGAL performance for AKI and CKD at readmission. Including unchanged pNGAL value as an increase in pNGAL (increased or unchanged delta-NGAL) didn’t create a much different performance

### CRP

Similar trends were observed using CRP as a marker for CKD and/or AKI instead of pNGAL, but all estimates were lower (Table 4). AUC for all CRP had a value of 0.70 for CKD and 0.78 for AKI (Fig. 5).

**Table 4:** CRP performance at optimal cutoff values

Kidney status	CRP cutoff (mg/L)	Sensitivity (%)	Specificity (%)	PPV (%)	NPV (%)
CKD admission	57	44 (27-61)	88 (83-93)	37 (22-52)	91 (87-95)
CKD readmission	4	65 (49-81)	46 (40-52)	13 (8-18)	91 (87-96)
AKI admission	16	74 (62-85)	77 (72-82)	37 (28-46)	94 (91-97)
AKI readmission	6	64 (52-77)	55 (51-58)	8 (6-11)	96 (94-98)

Receiver Operating Characteristic (ROC) were conducted to analyze CRP performance and calculate CRP cutoff value for AKI and CKD at admission and readmission

### Correlations between different variables

The correlation coefficient between pNGAL and CRP was 0.41 (95% CI: 0.34–0.48) for all patients and 0.43 (95% CI: 0.08–0.68) for patients with AKI at admission. Correlation between pNGAL and number of readmissions was 0.12 (95% CI: 0.06–0.19) for all patients and − 0.01 (95% CI: -0.28–0.27) for patients with AKI at readmission.

## Discussion

This prospective one-year follow-up study in an ED setting demonstrates that pNGAL is an excellent biomarker for ruling out CKD at admission. Moreover, our results did corroborate with our recent finding [37] that pNGAL also is an excellent AKI rule-out biomarker at admission in an ED.

### pNGAL prediction at admission

pNGAL showed good sensitivity for identifying standalone AKI and CKD at admission, highest for AKI. However, PPV values indicate many false positives. Specificity and NPV were both very good, making cutoffs more reliable for ruling out these conditions. The composite outcome had good sensitivity and specificity but low PPV for CKD or worse and AKI + CKD, limiting its usefulness. However, as AKI or worse had slightly better PPV with NPV similar to standalone conditions, composite cutoffs may likewise better indicate worse possible condition rather than diagnosis; non-AKI/non-CKD, not worse than AKI and not worse than CKD.

### pNGAL prediction at readmission

Prediction of standalone AKI and CKD at readmission had lower PPV than at admission, reducing usefulness even with the high sensitivity for CKD. Lower specificity limited cutoff usefulness for ruling out conditions, despite high NPV. This may be due to AKI/CKD requiring both susceptibility and exposure [5, 43], while NGAL reflects susceptibility and not exposure. Additionally, pNGAL, as an acute-phase reactant, spikes quickly and then normalize [12, 44].

### pNGAL cutoff values for AKI and CKD

When considering the estimated cutoffs, an increase in cutoff values for worsening conditions in the composite condition was observed, supporting the assumption of the conditions being considered as increasing in severity with relation to pNGAL. pNGAL cutoff values at admission varied by outcome. While standalone CKD and CKD or worse should yield the same cutoff, multiple cutoffs were found to have very similar Youden index values for both analyses, ranging from 83 to 158 ng/mL. This suggests no single cutoff for CKD at admission can clearly be considered superior. Instead, cutoffs can prioritize specificity and PPV (158 ng/mL) or favor sensitivity (81 ng/mL). Further, while cutoff values for CKD at admission and readmission were quite different, sensitivity and specificity were found to be similar for CKD at admission and readmission, at the same cutoff values. This along with the similar Youden index values across the scale for CKD at admission, suggest that the trend in sensitivity and specificity across different pNGAL cutoff is similar for CKD at admission and readmission, however specific optimal cutoffs, based on the Youden index differ.

For AKI the cases differ, as standalone AKI does not consider CKD whereas AKI or worse does. The larger cutoff value for standalone AKI is therefore likely a consequence of CKD only patients with pNGAL value below 111 ng/ml which in that analysis are considered only as ”not AKI”, contributing to a larger specificity for a higher cutoff value, again likely at a cost of the sensitivity.

All pNGAL cutoff values found for AKI are somewhat lower than those in our previous study (143 ng/mL) [37] and for both AKI and CKD compared to other studies done in an ED [22, 23, 25]. Some studies incorporate CKD by utilizing pre-admission laboratory results to support a potential diagnosis [22, 23, 25], while others acknowledge the data absence as a limitation [26].

Further, pNGAL cutoff values at admission were higher than at readmission for both AKI and CKD, reflecting untreated versus treated states. CKD had higher cutoffs than AKI due to distinct mechanisms: AKI causes a rapid NGAL surge from acute tubular damage, while CKD leads to gradual accumulation from impaired clearance. This distinction necessitates different detection thresholds.

Regarding the current study results for pNGALs prediction of CKD, they align with several studies. For instance, Bourgonje et al. [29] identified a significant association between pNGAL and the risk of developing CKD but found that it did not enhance CKD prediction for the general population beyond the standard eGFR measurement. However, the authors suggested that pNGAL could still hold potential in individuals with impaired kidney function or specific renal conditions. This might explain the findings of Danquah et al. [30], who reported high sensitivity and specificity for pNGAL in hypertensive patients (blood pressure ≥ 140/90), a group at high risk of renal failure.

Similar to our findings, a study also involving an ED population found increased pNGAL values for CKD compared to AKI patients. However, the predictive capability of pNGAL to distinguish between CKD and AKI patients was found to be limited [23]. Soto et al. [25] investigated pNGAL’s ability to predict CKD (stage ≥ 3) and while pNGAL was associated with a risk of eGFR decline, it did not significantly improve CKD prediction compared to other models.

Thus, while pNGAL alone cannot reliably distinguish between AKI and CKD, its diagnostic value may improve when interpreted alongside clinical history and complementary laboratory tests. A combined approach could therefore enhance differentiation between AKI and CKD. Recent studies support this direction: the eGFR-adjusted NGAL study [45] mentioned earlier improved AKI prediction by accounting for baseline kidney function, while findings from our recent published study [36], a subgroup of the current ED study population, demonstrated that integrating biomarkers such as pNGAL and CRP with established KDIGO risk factors significantly enhanced predictive performance. Together, these studies suggest that future diagnostic strategies should incorporate biomarker adjustment and multimodal risk assessment to achieve more accurate identification and stratification of kidney injury in heterogeneous ED populations.

### AKI to CKD transition and vise verse

It is well established that CKD increases the risk of AKI and vice versa [3, 46]. In this study, four patients with CKD at admission developed AKI, leading to simultaneous AKI and CKD (AKI in CKD) at readmission. However, our findings are too limited to draw conclusions about potential transitions between these conditions.

### Serial measurements of pNGAL

As one of the few studies [31–33] we examined serial changes in pNGAL but found that the predictive performance was lower for all parameter compared to the single pNGAL measures used in the other analyses, and therefore does not show any indication of being useful. We also examined variations in pNGAL during admission (supplementary Figs. 1 and 2); while pNGAL values were initially elevated in CKD patients, levels plateaued within a few days, and subsequent measurements revealed no distinct or consistent trend between groups.

### CRP in relation to pNGAL

The correlation coefficient of 0.41 suggests a weak correlation between CRP and pNGAL. When evaluating the performance of pNGAL and CRP for diagnosing AKI and CKD pNGAL showed better performance than CRP. However, its predictive accuracy for CKD at admission was only moderate but with relatively high specificity and NPV implying a good balance between over diagnosing and overseeing CKD.

### pNGAL versus uNGAL

In this study, only pNGAL was analyzed due to limited resources. While pNGAL can indicate kidney injury, it is as described earlier influenced by non-renal factors such as infection, inflammation, and heart failure [12, 47–49]. In contrast, urinary NGAL (uNGAL) more directly reflects tubular injury but is affected by urine flow and CKD. Studies report that pNGAL predicts AKI earlier [50, 51], whereas uNGAL better differentiates AKI types and outcomes [52]. However, variability among commercial assays and lack of standardization hinder consistent interpretation, and currently, only BioPorto’s PETIA test is FDA-approved and only for the pediatric AKI risk assessment indication [39].

### Strengths and limitations

This study benefits from a large cohort, a one-year prospective design, and access to prior-year data. Consecutive patient inclusion in an ED setting enhances generalizability. As limitations it is a single-center study, including only ED patients selected for blood testing and the high NPV values should be interpreted considering the large number of non-cases, which naturally inflates NPV. A key challenge in this studies NGAL measurements lies in the uncertainty about which molecular forms (monomeric, dimeric, or heterodimeric) and in what amounts they are detected by the assay. Additionally, 10% of patients (107/1,032) were excluded due to missing data, potentially introducing selection bias. For the serial NGAL analyses, only 29% of enrolled patients had more than one measurement, limiting inclusion in this subanalysis to this subset. Further, the study did not conduct stratified analysis by CKD stages, primarily due to the limited number of CKD cases. Stratification would result in subgroups with insufficient statistical power. Future studies with larger sample sizes could further explore the predictive performance of pNGAL across different CKD stages. Lastly, potential infectious or inflammatory conditions were not accounted for in this study, as our aim was to identify generalized pNGAL cutoff values applicable to a broad ED population rather than to control for all possible confounders. The analyses were designed to assess the predictive performance of pNGAL, not to explore associations, and therefore traditional confounding adjustments were not directly relevant. Additional variables (e.g., sex or comorbidities) were not included in the predictive models, as doing so would require multiple subgroup-specific cutoff values, which was beyond the scope of this study. Future studies aimed at developing comprehensive multivariable prediction algorithms for AKI and CKD could further explore the added predictive value of combining pNGAL with such factors.

## Conclusion

For predicting CKD in the ED, pNGAL proved to be an excellent biomarker for ruling out CKD at admission. Additionally, at readmission, pNGAL demonstrated strong utility for screening and excluding CKD. However, its limited diagnostic utility suggests that pNGAL alone should not be used to confirm CKD and must be complemented by other diagnostic tools.

pNGAL was observed to have a higher predictive performance than CRP for diagnosing both CKD and AKI at admission. Results were like those found in our previous study [37], supporting pNGAL as an excellent biomarker for ruling out AKI. Additionally, in the serial pNGAL measurements, CKD patients initially showed higher levels compared to non-CKD patients, but these differences appeared to diminish over time and did not clearly reflect changes in kidney function. Further, research is needed to better understand the risk factors for renal disease, stratify patients according to kidney function, and evaluate the effectiveness of using pNGAL as a primary diagnostic tool at ED admission.

## Electronic supplementary material

Below is the link to the electronic supplementary material.

Supplementary Material 1

## Data Availability

The study was approved by the Danish Data Protection Agency (VD-2019-143) and The Regional Committee on Health Research Ethics (H-19003424). Pseudonymized version of data may be requested by contacting the corresponding author Wetterstrand V. ( [vickywetterstrand@gmail.com](mailto: vickywetterstrand@gmail.com) ). Approval of the request from the Danish Data Protection Agency will however be needed before the data can be transferred; the corresponding author will assist with this request.
