# Supplementary material for: A longitudinal prospective study investigating serial measurements of plasma neutrophil gelatinase-associated lipocalins (NGAL) for the prediction of chronic kidney disease and acute kidney injury in emergency department patients
Source: BMC Nephrol. 2026 Jan 30;27:143. doi: 10.1186/s12882-026-04791-7 (PMC12930889; doi:10.1186/s12882-026-04791-7)
Supplement: Supplementary file 1 [file 12882_2026_4791_MOESM1_ESM.docx]

Supplementary figure 1. 

Graph over pNGAL levels at admission and the next 10 days for patients with CKD and non-CKD

The bold red and black lines represent the mean pNGAL over time for patients with CKD and those without CKD at admission, respectively.

Supplementary figure 2. 

Graph over pNGAL levels at admission and the next 10 days for all patients

The bold black line represents the mean value of pNGAL over time.
